# Deciphering the Potential of *Rhizobium pusense* MB-17a, a Plant Growth-Promoting Root Endophyte, and Functional Annotation of the Genes Involved in the Metabolic Pathway

**DOI:** 10.3389/fbioe.2020.617034

**Published:** 2021-01-18

**Authors:** Twinkle Chaudhary, Rajesh Gera, Pratyoosh Shukla

**Affiliations:** ^1^Enzyme Technology and Protein Bioinformatics Laboratory, Department of Microbiology, Maharshi Dayanand University, Rohtak, India; ^2^Department of Microbiology, Chaudhary Charan Singh Haryana Agricultural University, Hisar, India

**Keywords:** endophyte, plant growth-promoting rhizobacteria (PGPR), *Rhizobium pusense* MB-17a, ACC deaminase, *Vigna radiata*, siderophore, indole acetic acid, RAST

## Abstract

Plant growth-promoting rhizobacteria (PGPR) are root endophytic bacteria used for growth promotion, and they have broader applications in enhancing specific crop yield as a whole. In the present study, we have explored the potential of *Rhizobium pusense* MB-17a as an endophytic bacterium isolated from the roots of the mung bean (*Vigna radiata*) plant. Furthermore, this bacterium was sequenced and assembled to reveal its genomic potential associated with plant growth-promoting traits. Interestingly, the root endophyte *R. pusense* MB-17a showed all essential PGPR traits which were determined by biochemical and PGPR tests. It was noted that this root endophytic bacterium significantly produced siderophores, indole acetic acid (IAA), ammonia, and ACC deaminase and efficiently solubilized phosphate. The maximum IAA and ammonia produced were observed to be 110.5 and 81 μg/ml, respectively. Moreover, the PGPR potential of this endophytic bacterium was also confirmed by a pot experiment for mung bean (*V. radiata*), whose results show a substantial increase in the plant's fresh weight by 76.1% and dry weight by 76.5% on the 60th day after inoculation of *R. pusense* MB-17a. Also, there is a significant enhancement in the nodule number by 66.1% and nodule fresh weight by 162% at 45th day after inoculation with 100% field capacity after the inoculation of *R. pusense* MB-17a. Besides this, the functional genomic annotation of *R. pusense* MB-17a determined the presence of different proteins and transporters that are responsible for its stress tolerance and its plant growth-promoting properties. It was concluded that the unique presence of genes like *rpoH, otsAB*, and *clpB* enhances the symbiosis process during adverse conditions in this endophyte. Through Rapid Annotation using Subsystem Technology (RAST) analysis, the key genes involved in the production of siderophores, volatile compounds, indoles, nitrogenases, and amino acids were also predicted. In conclusion, the strain described in this study gives a novel idea of using such type of endophytes for improving plant growth-promoting traits under different stress conditions for sustainable agriculture.

## Introduction

The soil bacteria Rhizobia of the family Rhizobiaceae cover an array of different bacterial genera, including *Sinorhizobium, Rhizobium, Mesorhizobium, Bradyrhizobium, Azorhizobium*, and *Allorhizobium* (Manasa et al., 2017). The symbiotic association between the soil bacterium *Rhizobium* and leguminous crops was first reported by Frank (1889) in stem- and root-nodulating bacteria. Generally, most of the rhizobial species are endophytes and colonize intracellularly for root growth promotion (Mus et al., 2016). Rhizobia can promote plant growth by both direct and indirect methods (Gopalakrishnan et al., 2015). Furthermore, many rhizobial species such as *Rhizobium oryziradicis, Rhizobium rhizoryzae, Rhizobium oryzicola, Rhizobium pseudoryzae*, and *Rhizobium rhizosphaerae* show a non-symbiotic association with paddy crops (Zhang et al., 2015; Zhao et al., 2017). Rhizobia is also employed as plant growth-promoting rhizobacteria (PGPR) to increase plant growth through phosphate solubilization, fixation of nitrogen, development of ACC deaminase, siderophore development, and production of indole-3-acetic acid (IAA) (Pravin et al., 2016). This symbiosis relationship between the rhizobium and leguminous crop is essential for better productivity in agricultural systems. It was reported that merely 2–3% of bacteria help in root growth promotion (Souza et al., 2015). The rhizospheric zone is the core and chief region of microbiome interaction with its host plant due to the presence of food exudates. Rhizobium is well-known for plant microbiome due to its growth-promoting traits (Poole et al., 2018; Ferreira et al., 2019). Among all rhizobial species, *Bradyrhizobium japonicum* is reported as the best one for siderophore production and *Rhizobium leguminosarum trifolii* for phosphate solubilization and IAA production on a large scale (Garcia-Fraile et al., 2012). Hence, the isolation, selection, screening, and exploitation of efficient endophytic bacteria in agricultural practices have immense significance in maintaining the sustainability of agroecosystems.

On the other hand, an *in silico* study of various strains revealed which types of genes and how many are responsible for the plant growth-promoting (PGP) traits. It is reported that *Bacillus subtilis* Marbug 168 was the first PGPR strain that was sequenced and analyzed. It is composed of 4,100 genes that code for different proteins (Moszer, 1998). Along with this, a genomic study of other rhizobial species and PGPR strains that act as biofertilizers show that they play a significant role in bio-inoculant technology because they help in the identification of those genes that have an invaluable role and beneficial activity in agriculture (Ayuso-Calles et al., 2020).

Therefore, the overarching goals of the present study include (1) the isolation and identification of the *Rhizobium* isolated from the roots of *Vigna radiata*; (2) the characterization of its PGPR traits such as siderophore production, ammonia production, IAA production, HCN, ACC deaminase enzymatic activity, and *V. radiata* plant growth enhancement by pot experiment; and (3) *in silico* analysis by genome annotation to find out the particular functions carried by different genes.

## Materials and Methods

### Bacterial Culture

The bacterial culture used for this study was procured from the Department of Microbiology, CCS Haryana Agricultural University, Hisar, India, and stored on yeast extract mannitol agar (YEMA) media plates (Hossain et al., 2019). The abiotic stress tolerance, PGPR properties, phylogenetic analysis, and genomic annotation were then studied for further analysis.

### Effects of Abiotic Stress Factors on *Rhizobium pusense* MB-17a

#### Temperature Tolerance

**Figure 1:**
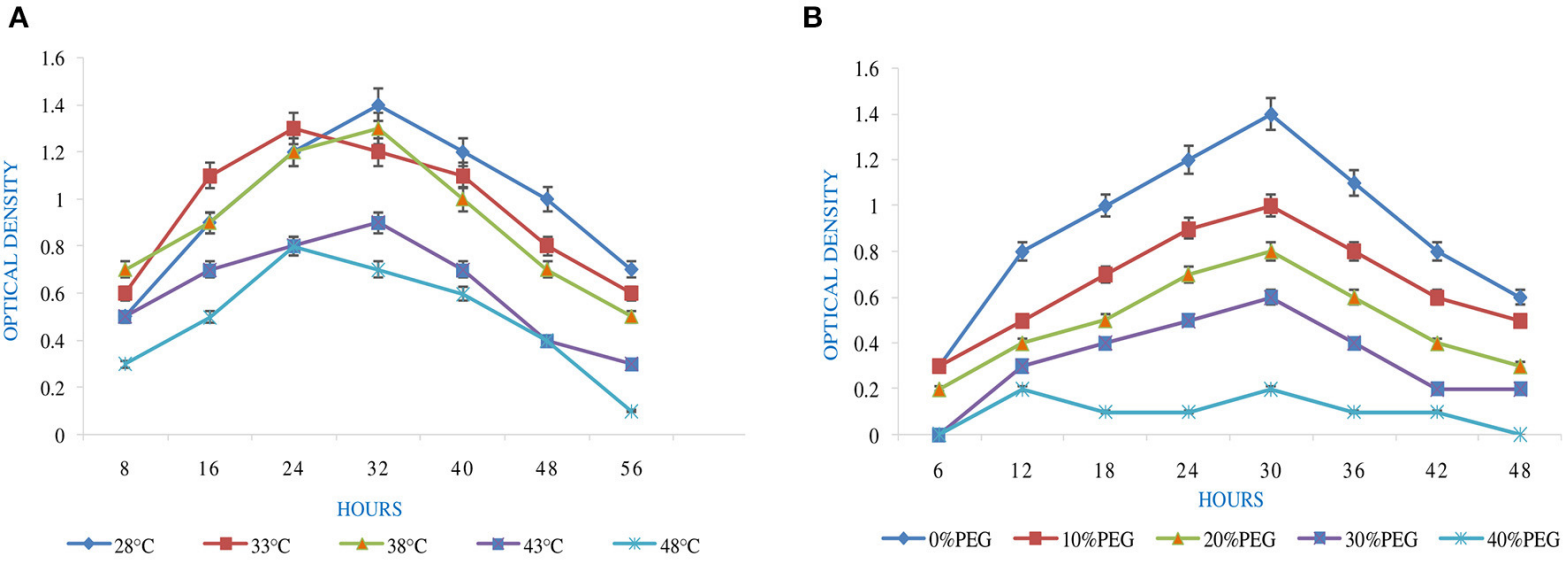
Effect of abiotic factors **(A)** temperature and **(B)** PEG on the growth of *Rhizobium pusense* MB-17a.

Temperature tolerance analysis was deliberated with five sets of temperature, i.e., 28 to 53°C (Figure 1A). This was achieved by inoculating the isolate in YEMA broth and incubating it at 110 rpm for 56 h by taking their generation time. The resulting growth was observed at 600 nm against the sterile medium by using a spectrophotometer (Igiehon et al., 2019).

#### High pH and Salt Tolerance

The optimum and favorable range of pH was determined by inoculating the isolate in YEMA broth at different pH ranges (3.0 to 11) (Table 1). The inoculated culture flask was incubated for 48 h at 110 rpm and 28°C by taking their generation time. Finally, the growth of the isolate was observed at 600 nm by taking an uninoculated culture as a blank (Hang et al., 2019; Tewari and Sharma, 2020). In the same way, salt tolerance was observed by adding different concentrations of NaCl, i.e., 100 to 400 mM, into the YEMA broth. The inoculated culture flask was incubated for 48 h at 110 rpm and 28°C. Finally, the growth of the isolate was observed at 600 nm by taking an uninoculated culture as blank (Mrabet et al., 2011).

**Table 1:** Different buffer systems for the pH tolerance test.

**S. No**.	**Buffers used**	**pH range**
1	Citrate buffer	3
2	Phosphate hydroxide buffer	5
3	Tris–HCl buffer	7
4	Carbonate hydroxide buffer	9
5	Phosphate hydroxide buffer	11
6	Chloride hydroxide buffer	13

#### Polyethylene Glycol (PEG) as Drought Stress

Drought tolerance was determined by inoculating the rhizobial isolate in YEMA broth with different ranges of PEG 6000, i.e., 0, 10, 20, 30, and 40% (Yadav et al., 2015; Mondal et al., 2017). The inoculated flasks were incubated for 48 h at 110 rpm and 28°C by taking their generation time. Finally, the growth of the isolate was observed at 600 nm by taking an uninoculated culture as a blank. Each experiment of abiotic stress tolerance was done in triplicate.

### Biochemical and Morphological Characterization

The morphological features of the test isolate were characterized by using a Gram staining protocol. Besides, biochemical tests like Methyl Red and Voges-Proskauer (MRVP), indole, catalase, citrate utilization, and oxidase tests were performed (Rana et al., 2011; Andy et al., 2020).

### Evaluation for Different PGP Traits

#### Phosphate Solubilization Activity

##### Qualitative Screening for PSB

The bacterial isolate was screened for phosphate solubilization activity by a qualitative method on a Pikovskaya (1948) agar medium plate containing Ca_3_ (PO_4_)_2_. For this, the bacterial isolate was spotted on the Pikovskaya agar plate and incubated at 28°C for 7 days (Mehta and Nautiyal, 2001). The occurrence of the halo zone around the spotted culture specifies phosphate solubilization activity. The halo zone formed around the colony was determined by the phosphate solubilization index (PSI). It was calculated as the ratio of the total diameter (zone **+** colony) to the colony diameter (Premono et al., 1996; Chawngthu et al., 2020):

(1) $$\begin{array}{r} PSI=\frac{Colony\;diameter+Halo\;zone\;diameter}{Colony\;diameter} \end{array}$$

##### Quantitative Screening for PSB

The stannous chloride method was used for the quantitative screening of solubilized phosphate (King, 1932). For the solubilization activity, the bacterial isolate was inoculated in the National Botanical Research Institute (NBRIP) liquid media containing tricalcium phosphate and incubated at 28°C for 2–10 days at 110 rpm. The culture was centrifuged for 20 min at 4°C and 8,000 rpm. Mixed with 100 μl of supernatant was 3.2 ml of distilled water, accompanied by 500 μl of molybdate solutions. In addition, 200 μl of stannous chloride solutions was mixed with this solution and vortexed for 5 min. Finally, the sample was incubated for 40 min at room temperature, and optical density was measured at 600 nm. The quantity of phosphate solubilization was measured by using the KH_2_PO_4_ standard curve (Olsen and Sommers, 1982).

#### Siderophore Production

For the qualitative assay of the siderophore production, the bacterial culture was spotted on Chrome Azurol S (CAS) agar medium plate. The spotted CAS agar plate was incubated at 28°C for 6 days (Schwyn and Neilands, 1987). After incubation, a clear orange-yellow halo zone around the spotted culture indicates a positive result for siderophore production.

#### HCN Production

The protocol mentioned by Kremer and Souissi (2001) was used to evaluate HCN output. For this, on a King's B agar plate supplemented with 4.4 g of glycine per liter, the bacterial isolate was streaked. After that, in the picric acid solution (0.05% solution in 2% sodium carbonate), a Whatman No. 1 filter paper was dipped and placed on the cover of the streaked plate. The streaked plate was covered by Parafilm and incubated at 28°C for 5 days. The change in the color of the lid from yellow to orange-brown shows a positive result for HCN production (Ali et al., 2020).

#### Ammonia Production

A qualitative assay for NH_3_ production was carried out by using the protocol of Cappucina and Sherman. In this method, 72 h-old bacterial culture was inoculated in 10 ml of peptone water and incubated at 28°C for 3 days. Subsequently, 1 ml of Nessler's reagent was added to each inoculated tube. The appearance of a yellow-brown color shows the positive result of ammonia production (Cappuccino and Sherman, 1992). The resulting ammonia production was quantified and measured by a spectrophotometer at 450 nm by using 0.1–10 μmol ammonium sulfate standard curve.

#### IAA Production

The IAA production of the rhizobial isolate was determined by the colorimetric assay described by Tang and Bonner (1948). The isolate was inoculated in YEM broth supplemented with 100 to 500 μg/ml of l-tryptophan and placed at 28°C for 2–7 days. The culture was then centrifuged at 10,000 *g* for 12 min. The supernatant was mixed with Salkowski reagent, and the IAA concentration was determined. The occurrence of a light pink color indicates the positive result for IAA production. The total amount of IAA was estimated by using the standard curve of known IAA concentration (10–200 μg/ml). Negative control was taken by mixing 2 ml of sterile broth with 2 ml of Salkowski reagent. This experiment was done in triplicate, and average reading was taken (Gordon and Weber, 1951).

#### 1-Aminocyclopropane-1-Carboxylate Deaminase (ACCD) Production

To determine the ACC deaminase activity, the selected isolate was inoculated in 10 ml of YEM broth at 28°C up to their stationary growth phase. For the inducible activity of ACC deaminase, the cultured cells were centrifuged and washed with 0.1 M of Tris–HCl at pH 7.5. After this, the cells were suspended in 4 ml of DF minimal medium that was supplemented with 3 mM of ACC and incubated at 30°C at 120 rpm for 72 h.

After optimum growth, ACC deaminase activity was measured by calculating ammonia and α-ketobutyrate concentration by the breakdown of ACC through ACC deaminase (Penrose and Glick, 2003). The highly induced bacterial cells were collected by the centrifugation process at 4,000 *g* for 4 min. Afterward, the cells were washed with 0.1 M of Tris–HCl at pH 7.5 and again suspended in 200 μl of 0.1 M Tris–HCl at pH 8.5. For 40 s, the resulting cells were mixed with toluene, then vortexed, and incubated at a temperature of 28°C for 30 min with 5 μl of 0.3 M ACC. The negative control for this test was 50 μl of bacterial body cell suspension without ACC while the blank had 50 μl of 0.1 M Tris–HCl with 5 μl of 0.3 M ACC at pH 8.5. The resulting sample was mixed with 500 μl of 0.5 N HCl, and at 10,000 *g* centrifugation, cell debris was removed. A 1-ml sample supernatant was mixed with 500 μl of 0.5 N HCl and 200 μl of dinitrophenylhydrazine (2,4DNPH) solutions and incubated at 30°C for 30 min. After this process, 1 ml of 2 N NaOH was mixed with the prepared sample, and absorbance was taken at 540 nm. The resulting sample was compared with the standard curve of α-ketobutyrate ranging between 0.1 and 1.0 μmol.

#### Antibiotics Sensitivity Test

For this test, the standard disk diffusion method was used to determine the antibiotics sensitivity of selected rhizobial isolates (Massa et al., 2020). The rhizobial culture was grown and spread on YEMA media plates and incubated for 72 h at 28°C. Different HiMedia antibiotic disks were used: kanamycin (30 μg), nalidixic acid (30 μg), cefotaxime (30 μg), penicillin G (10 μg), and cefotaxime (30 μg). These discs were placed on the YEMA plates with their different concentrations, and the inhibition zone was deliberated in mm around the disk.

#### Antifungal Activity

The selected isolate MB-17a was screened for antifungal activity against *Fusarium oxysporum* by using PDA according to the method of Dikin et al. (2006). The fungal culture was taken from the PDA plate and placed at the center of the YEMA plate that was inoculated by the selected bacterial isolate. This antifungal activity was measured after 5 days, and the zone of inhibition was measured by the following formula: % Inhibition = Radial growth of the pathogen in control (mm)—Radial growth of the pathogen in treatment (mm)/Radial growth of the pathogen in control (mm).

### Identification of PGPR Strain by 16S rRNA Gene Sequencing and Its Phylogenetic Analysis

The genomic DNA of the bacterial isolate was extracted by the phenol/chloroform CTAB method (Wilson, 2001). The extracted DNA was used to set up a PCR for the amplification of the 16S rDNA gene with specific primers using 16S forward and 16S reverse primers (Hakim et al., 2020). The amplification of a ~1,500 bp amplicon was observed. The eluted PCR product was sequenced by using an ABI 3730xl sequencer by Sanger's method. The obtained data were compared and analyzed by using BLAST on the National Center for Biotechnology Information (NCBI). The sequences obtained were submitted to the NCBI gene bank, and the isolate accession numbers were assigned. By using Clustal W software, the phylogenetic tree was constructed by the neighbor-joining method (Ji et al., 2020).

### Pot Experiment

An experiment with pot culture at CCS HAU, Hisar, India, has determined the impact of *Rhizobium pusense* MB-17a isolate on the growth of *V. radiata*. This triplicate experiment was performed using a fully randomized design (RCBD). The crop was grown in 8-in. earthen pots in 5 kg of non-sterile soil taken from the fields of CCS, HAU (longitude: 75°46′E and latitude: 29°10′N). With different field capacities, i.e., 50 and 100% FC, each pot was irrigated regularly. The recommended fertilizer doses, i.e., NPK (potassium muriate, phosphate urea, and urea were added at a ratio of 10:70:27/kg/ha before sowing the crop. *V. radiata* seeds were surface-sterilized by dipping in 95% ethanol, then immersed for 4 min in 0.2% mercuric chloride, and washed eight times with distilled water. On the other hand, the bacterial culture was grown in YEMA broth and incubated at 30°C. By taking absorbance at 600 nm (10^9^-10^10^ cells/ml), 1 ml of this overnight grown culture was applied on each *V. radiata* seed for 15 min followed by the sowing of seeds to a depth of 5 mm. In the control pot, seeds were treated with only the YEMA medium, not with the culture suspension. In contrast, seeds of *V. radiata* were also inoculated as a reference bacterial strain, i.e., *Rhizobium pusense* MB-703, for the comparison with *Rhizobium pusense* MB-17a.

All the pots were placed in sunlight and regularly irrigated according to their field capacity. Each set of experiments was harvested after 30, 45, and 60 DAS. At each interval, plant dry weight, plant fresh weight, shoot length, root length, nodule number, nodule fresh weight, and nodule dry weight were measured.

### Statistical Analysis

All these pot experiments were performed in triplicate (*n* = 3), and the data were expressed as the mean ± SD. Values were analyzed by using one-way ANOVA, and a *p* < 0.05 was considered statistically significant.

### *In silico* Analysis

*R. pusense* MB-17a genomic sequence was retrieved on the website of NCBI (https://www.ncbi.nlm.nih.gov/nuccore/NC_022545.1). The genome annotation analysis for PGP traits was performed by using the Rapid Annotation using Subsystem Technology (RAST), online software (Aziz et al., 2008). Besides, SEED viewer methods in the SEED website were used to conduct a practical genome analysis (Overbeek et al., 2014; Zinina et al., 2019). The data analysis and genomic annotation for the *R. pusense* MB-17a strain contributes to the clarification of its extraordinary taxonomic classification and its adequacy for large genome studies (Suarez et al., 2019). On the whole, the genome generally supplies information to validate, detect, and understand many of its previously assessed features that are relevant to the promotion of plant growth under various stress conditions.

## Result and Discussion

### Biochemical and Morphological Characterization

The MB-17a strain was a Gram-negative, small rod-shaped strain that produces a white milky colony with a smooth surface on the YEMA agar plate. The results for the development of indole, catalase, and nitrate reductase were shown to be positive. Citrate could also be used as a source of carbon. Other test results for H_2_S production, oxidase test, and Voges-Proskauer were reported to be negative by MB-17a (Table 2).

**Table 2:** Biochemical and morphological characterization of isolate MB-17a.

**S. No**.	**Characteristic**	**Activity**
**Morphology**		
1	Gram reaction	**–**
2	Shape	Rods
**Biochemical reaction**		
3	Citrate utilization	**+**
4	Voges-Proskauer	**–**
5	Methyl Red	**+**
6	Indole	**+**
7	catalase test	**+**
8	H_2_S production	**–**
9	Nitrate reductase	**+**
10	oxidase test	**–**
**Carbohydrate utilization**		
11	Sucrose	**–**
12	Mannitol	**+**
13	Lactose	**–**
14	Dextrose	**–**
15	Hydrolysis	**+**
16	Gelatin	**–**
17	Starch	**+**
**Enzyme production**		
18	Cellulase	**+**
19	Chitinase	**+**
20	Protease	**+**
21	Glucanase	**+**
**Antibiotic susceptibility test**		
22	Kanamycin	R^*^
23	Nalidixic acid	R^*^
24	Cefotaxime	R^*^
25	Penicillin G	R^*^
26	Cefotaxime	R^*^

* *R, Resistant*.

### Effect of Abiotic Stress Factors on *Rhizobium pusense* MB-17a

**Figure 2:**
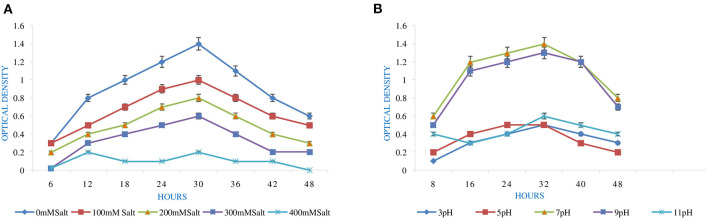
Effect of abiotic factors **(A)** salt and **(B)** pH on the growth of *Rhizobium pusense* MB-17a.

pH is an essential factor that affects the growth of microbes to a large extent. Initially, the bacterial isolate MB-17a shows little growth at pH 3, but after attaining pH from 5 to 9, it showed significant growth. It showed that growth increased with a rise in pH, accompanied by a decrease in alkaline pH (Msimbira and Smith, 2020). The effect of different pH values on the growth of *Rhizobium pusense* MB-17a is demonstrated in Figure 2B. Along with pH, salt concentration also affects the growth of microbial strain on a large scale. Sometimes, the symbiosis process between the rhizobial and its host plant enables it to tolerate the salt concentration to a large extent (Abdiev et al., 2019). In the present study, it is observed that MB-17a showed optimum growth up to 100 mM salt concentration, but after a high salt concentration, i.e., from 200 to 400 mM, its growth decreases with an increase in salt concentration (Figure 2A). Like salt and pH, temperature is the most significant factor that influences the growth of microorganisms. It was reported initially that the rhizobial strain shows optimum growth at temperatures of 28 to 33°C. After this range, their activity becomes low and shows no growth (Alexandre and Oliveira, 2013). In this study, *R. pusense* MB-17a has also shown the most favorable growth at 28 and 33°C, but after this range, the growth of the strain decreases with a high rate, and at the last range, it showed no growth at 53°C, as shown in Figure 1A. Another abiotic factor, i.e., PEG concentration, also affects the growth of the microbial strain (Kour et al., 2020). Optimum growth was observed up to 10% PEG concentration, but after this range, the growth of MB-17a declined to a large extent (Figure 1B). This shows that MB-17a can adapt up to a limited range of stress and can enhance the growth of the host plant.

### IAA and ACCD Production

**Figure 3:**
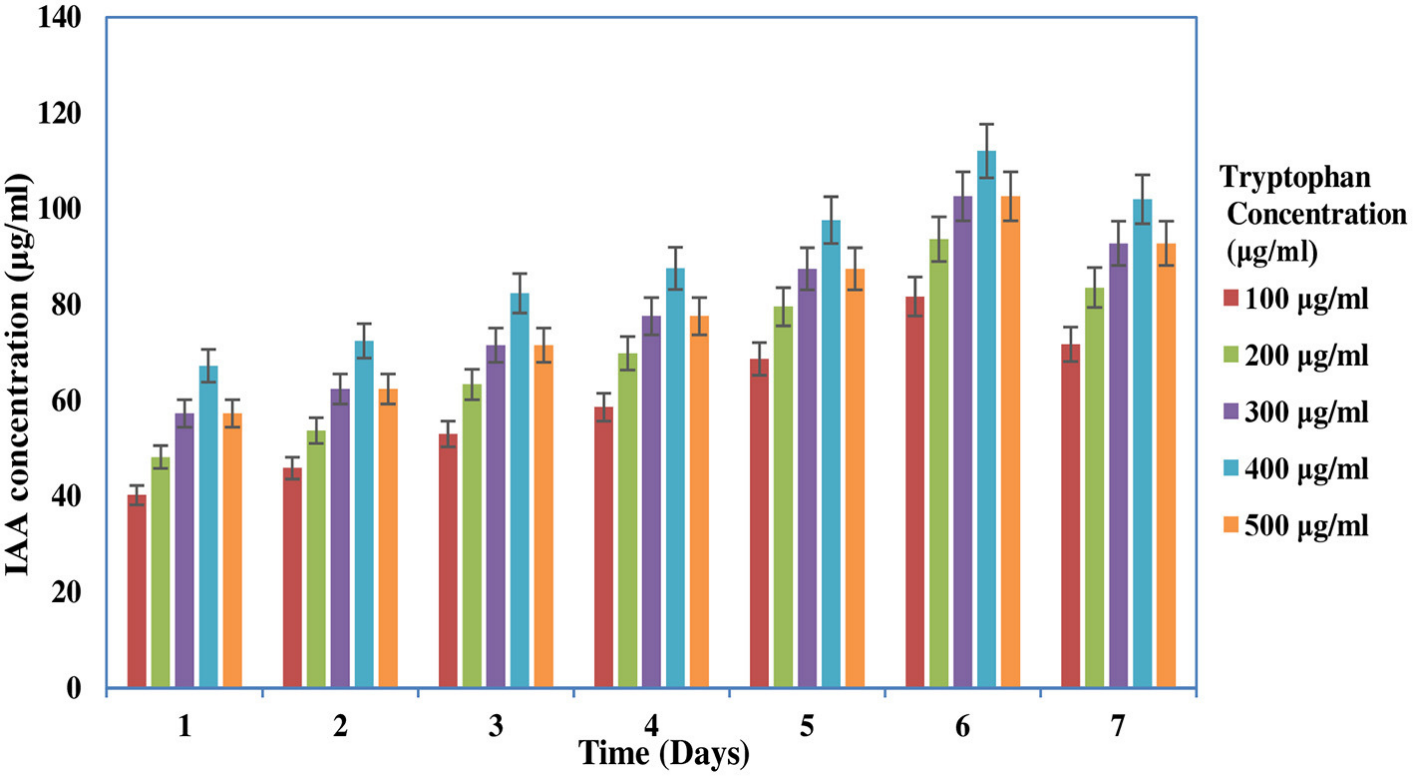
Effect of time and different tryptophan concentrations on IAA production.

The screening of PGP traits of MB-17a is shown in Table 3. The significant attribute of this isolate is the production of indolic phytohormonal compounds. These phytohormones encourage the root length and root growth on a large scale, which helps the host plant take more nutrients from the soil (de Souza et al., 2013). This bacterial strain exhibited better IAA-producing capability and produced IAA of 15.5 μg/ml. It is observed that IAA production was more increased in the presence of tryptophan and showed various levels in the different concentrations of tryptophan. This isolate showed a high quantity of IAA production with an increase in tryptophan concentration up to 450 μg/ml (Figure 3). The maximum IAA production by this isolate is 110.5 μg/ml (Table 3). In various studies, it is reported that several rhizobial species enhance IAA and auxin production in response to l-tryptophan during salinity and stress conditions (Iqbal et al., 2016). Lebrazi et al. (2020) reported a production of 116 and 105 μg/ml IAA by *Rhizobium* species.

**Table 3:** Assessment of PGP traits of strain MB-17a.

**PGP traits**	**Results**
IAA production	110.5 μg/ml
Phosphate solubilization	53 μg/ml
Ammonia production	81 μg/ml
alpha-Ketobutyrate production	18 μM/mg protein/h
Siderophore production	**+**
Antagonistic activity	**+**
HCN production	**+**

**Figure 4:**
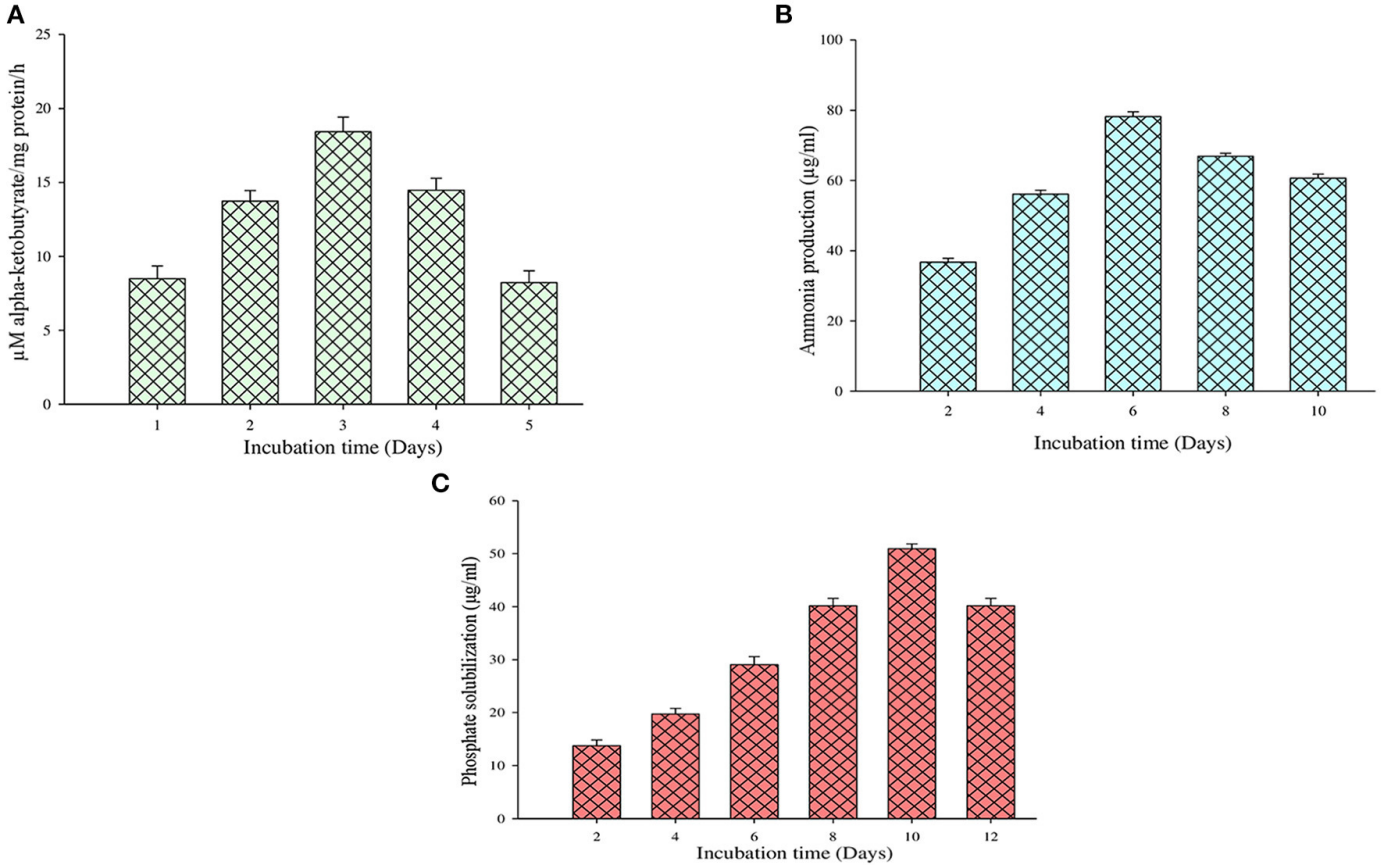
Quantification evaluation of **(A)** ACC deaminase activity, **(B)** ammonia production, and **(C)** phosphate solubilization.

On the other hand, the ACC deaminase enzyme activity was determined by quantifying the α-ketobutyrate amount during the breakdown of ACC by the ACC deaminase enzyme (Figure 4A). It is observed that during this test, the MB-17a isolate uses ACC as a nitrogen source by synthesizing ACC deaminase enzyme, which results in 18 μM/mg protein/h alpha-ketobutyrate production (Table 3).

### Ammonia Production and Phosphate Production

The other significant PGP trait of this isolate is ammonia production. By this trait, the selected isolate reported ammonia production by utilizing peptone as a substrate which is used by the host plant. Sometimes, large ammonia production also generates alkaline conditions in the soil, which checks the growth of various pathogenic fungi (Karthika et al., 2020). The study shows that 81 μg/ml of ammonia was produced by the MB-17a isolate (Figure 4B).

The selected isolate showed a significant zone of phosphate solubilization on the Pikovskaya agar medium plate containing Ca_3_ (PO_4_)_2_. A clear zone appeared around the colony after 6 days of incubation. The PSI of this isolate was 2.67, and in quantitative measurement, it showed a significant phosphate solubilization value of 53 μg/ml (Figure 4C).

### Determination of Antibiotics Sensitivity Test and Antifungal Activity

The selected isolate showed resistance against kanamycin, nalidixic acid, cefotaxime, penicillin G, and cefotaxime antibiotics. It was observed that it showed 2.4, 3.1, 4.1, 3.5, and 4.2 mm zones of inhibition against kanamycin, nalidixic acid, cefotaxime, penicillin G, and cefotaxime, respectively. Hence, it showed that this isolate possessed good resistance against different types of antibiotics.

The selected isolate showed a significant positive result for the antifungal activity. It showed a zone of inhibition of 18 mm against the test pathogen. It is observed that this isolate produces enzymes like cellulases, chitinases, proteases, and glucanases that help in the cell wall degradation of fungi in the culture plate. Yang et al. (2019) reported that along with rhizobia, many other PGPR isolates exhibit this antifungal activity against fungus-like *Phytophthora parasitica, Phytophthora capsici* and *F. oxysporum*.

### Determination of HCN and Siderophore Production

The HCN-producing ability of this isolate was confirmed on a King's B agar medium plate. It showed an orange-brown color on the lid of the plate that shows a positive result for HCN production. It is reported that HCN plays a significant role in checking the growth of pathogenic fungi in soil. On the other hand, the siderophore-producing ability of this isolate was confirmed by a CAS agar plate assay. This isolate produced a significant orange halo zone around the inoculated isolate on the CAS agar plate media (Table 3).

### 16S rRNA Sequencing and Its Phylogenetic Analysis

**Figure 5:**
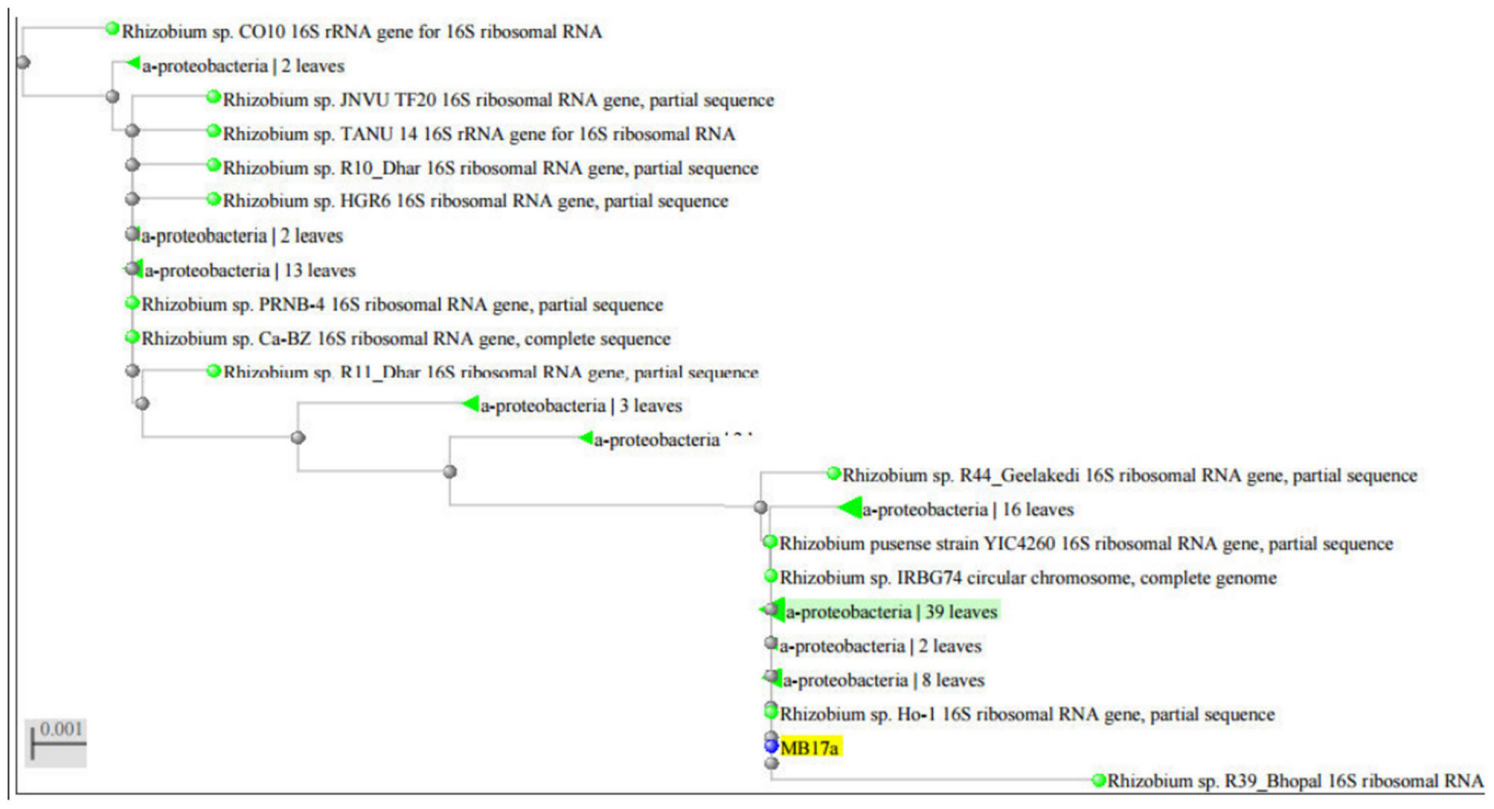
Phylogenetic tree of *Rhizobium pusense* MB-17a with their closest neighbor.

The strain MB-17a was identified on the basis of the 16 rRNA sequences with gene bank accession number MW021471, which belonged to *R. pusense*. There was 100% sequence similarity with *R. pusense* strain YIC4260, *Rhizobium* species IRBG74, *Rhizobium* species JNVUTL9, *Rhizobium* species R55, *Rhizobium* species HGR13, *Rhizobium* species CO51, *Rhizobium* species Ho-1, *Rhizobium* species LM-5. Furthermore, it has 99% sequence similarity with *Rhizobium* species DG22. The phylogenetic tree of *Rhizobium pusense* MB-17a with its closest neighbor strain is constructed (Figure 5).

### Assessment of PGP Traits in Response to Plant Growth Promotion

The potential of the *Rhizobium pusense* MB-17a for plant growth was determined by using as bio-inoculant for the *V. radiata* crop by performing a pot experiment. A reference strain MB-703 was taken for comparison purposes with the test inoculant. The obtained data revealed that *Rhizobium pusense* MB-17a showed stimulatory effects on plant weight, plant height, nodule number, nodule weight, shoot length, and root length. There was a substantial increase in the plant's fresh weight by 76.1% and dry weight by 76.5% at the 60th day after inoculation (Table 4). Also, there is a significant increase in the nodule number by 66.1% and nodule fresh weight by 162% at the 45th day after inoculation with 100% field capacity after the inoculation of *R. pusense* MB-17a (Table 6). Furthermore, it also showed a positive impact on shoot length and root length with 52.8% and 64.3% enhancement after 60th DAS (Table 5). The changes in each parameter are shown in Tables 4–6 at regular intervals, i.e., after 30, 45, and 60 DAS. Hence, the *Rhizobium pusense* MB-17a was found to be more effective and beneficial for plant growth promotion. These stimulatory results of *Rhizobium pusense* MB-17a are responsible for its diazotrophic nature, and therefore, it can be considered a PGPR strain for plant growth promotion. A similar type of results and increase in biomass has been studied by many researchers by taking into account the many endophytic rhizobia-like families such Phyllobacteriaceae, Rhizobiaceae, and Bradyrhizobiaceae (Lugtenberg and Kamilova, 2009; Bhattacharyya and Jha, 2012). They show a unique association potential with the root nodules of leguminous plants. Mehboob et al. (2012) also studied the potential of rhizobia with some non-leguminous plants.

**Table 4:** Effect of *Rhizobium pusense* MB-17a on plant fresh weight and dry weight on *Vigna radiata* growth at the 30th, 45th, and 60th DAS.

**Treatment**	Plant fresh weight (g^*^)			Plant dried weight (g^*^)		
	**30th DAS**	**45th DAS**	**60th DAS**	**30th DAS**	**45th DAS**	**60th DAS**
Control	6.56 ± 0.98	9.63 ± 1.24	10.5 ± 0.85	1.9 ± 0.45	4.06 ± 0.54	4.76 ± 0.36
RDF	7.53 ± 0.65	10.3 ± 0.86	12.5 ± 0.89	2.96 ± 0.49	4.53 ± 0.62	6.33 ± 0.65
RDF + MB-17a (50% FC)	8.7 ± 0.74	12.6 ± 0.94	15.56 ± 0.74	3.76 ± 0.44	5.9 ± 0.24	7.36 ± 0.62
RDF + MB-17a (100% FC)	10.6 ± 0.77	15.4 ± 0.82	18.56 ± 0.65	4.93 ± 0.498	6.1 ± 0.29	8.3 ± 0.94

* *Average values of triplicates (mean ± SE)*.

**Table 5:** Effect of *Rhizobium pusense* MB-17a on shoot and root length on *Vigna radiata* growth at the 30th, 45th, and 60th DAS.

**Treatment**	Shoot length (cm^*^)			Root length (cm^*^)		
	**30th DAS**	**45th DAS**	**60th DAS**	**30th DAS**	**45th DAS**	**60th DAS**
Control	13.66 ± 0.93	20.7 ± 0.98	22.7 ± 0.86	8.4 ± 0.89	14.6 ± 0.85	15.7 ± 0.98
RDF	14.7 ± 0.73	20.86 ± 1.34	24.66 ± 0.85	10.4 ± 0.75	15.5 ± 0.61	17.6 ± 1.02
RDF + MB-17a (50% FC)	18.66 ± 0.93	28.33 ± 1.03	32.63 ± 0.93	11.6 ± 0.74	16.5 ± 0.65	23.53 ± 0.93
RDF + MB-17a (100% FC)	19.63 ± 0.81	30.66 ± 0.93	34.7 ± 0.81	13.6 ± 0.82	17.4 ± 0.77	25.8 ± 0.81

* *Average values of triplicates (mean ± SE)*.

**Table 6:** Effect of *Rhizobium pusense* MB-17a on nodule number and nodule dry and fresh weight on *Vigna radiata* growth at the 30th and 45th DAS.

**Treatment**	Number of nodule^*^		Fresh weight nodule (mg^*^)		Dry weight nodule (mg^*^)	
	**45th DAS**	**60th DAS**	**45th DAS**	**60th DAS**	**45th DAS**	**60th DAS**
Control	30.33 ± 1.69	13 ± 0.81	199.06 ± 1.11	98.8 ± 0.59	89.53 ± 0.81	51.2 ± 1.88
RDF	35.33 ± 1.24	15.66 ± 0.94	208.46 ± 0.81	106.46 ± 0.77	94.66 ± 4.69	53.5 ± 0.74
RDF + MB-17a (50% FC)	46.33 ± 1.24	18 ± 0.81	495.56 ± 0.83	248.73 ± 0.94	178.76 ± 1.37	94.33 ± 1.02
RDF + MB-17a (100% FC)	51.66 ± 2.05	21.66 ± 2.05	511.43 ± 0.77	257.5 ± 1.71	189.5 ± 0.97	96.46 ± 0.61

* *Average values of triplicates (mean ± SE)*.

### *In silico* Analysis

**Figure 6:**
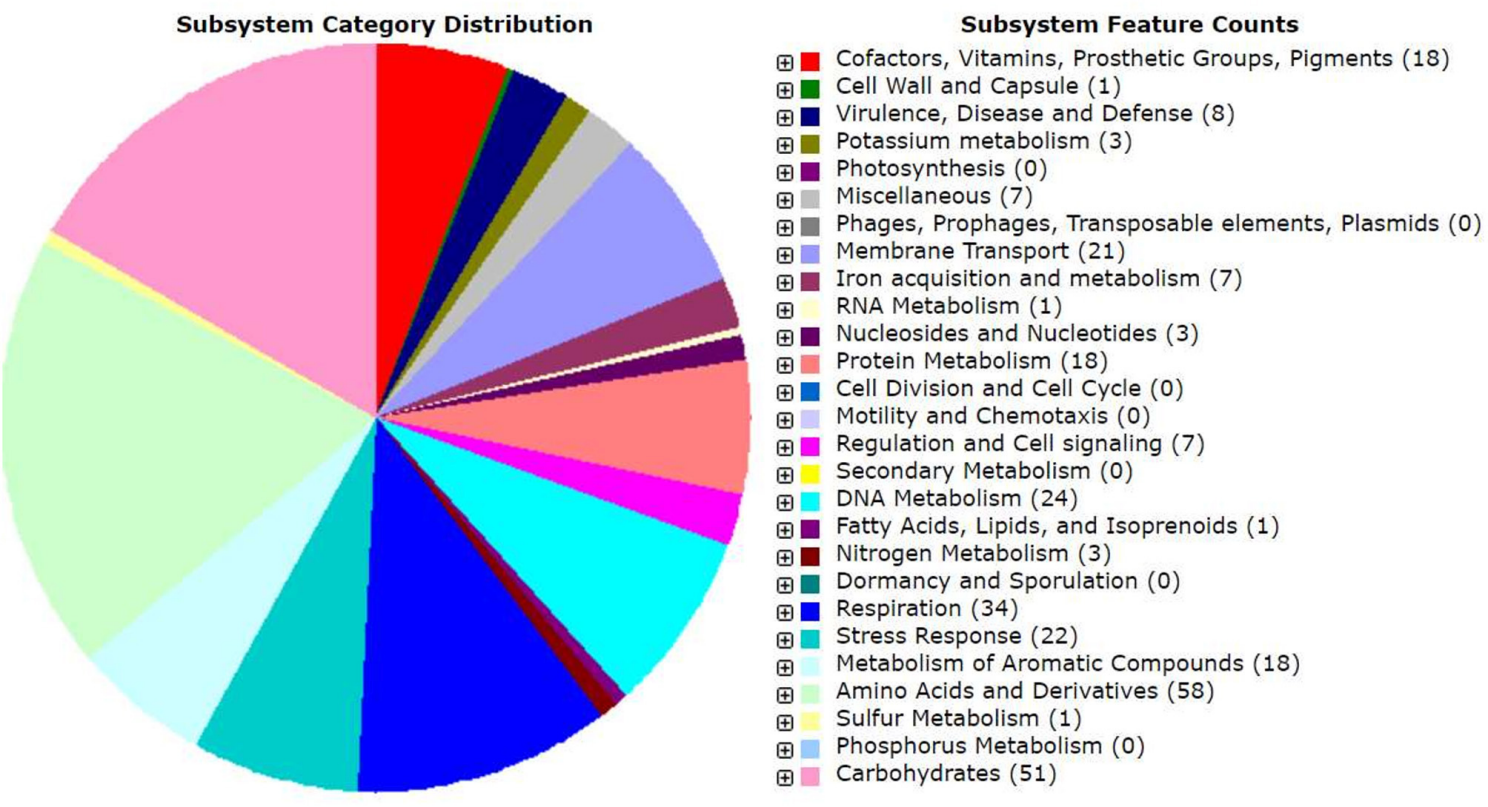
Genomic annotation of *Rhizobium pusense* MB-17a.

Genomic annotation of the *R. pusense* MB-17a strain was performed to know the PGP traits and genes responsible for various stress tolerance factors. In general, the annotated genome usually gives information to validate, identify, and recognize many of the species' previously assessed properties that are significant to the promotion of plant growth under various stress conditions. It is observed that this species has many other genes that carry out different particular functions like sulfur, protein, and RNA metabolism; capsule and cell wall formation; potassium and nitrogen metabolism; iron acquisition; defense, disease, and virulence mechanisms; membrane transport; stress response; metabolism of aromatic compounds; cell signaling regulation; regulation of pigments; and vitamins and prosthetic group formation (Figure 6). However, this study is focused on the genes that are responsible for iron acquisition and its metabolism, nitrogen metabolism, membrane transport, stress response, defense and virulence mechanisms, and cell signaling that marked its potential for PGPR traits (Table 7). In this study, the genome of *R. pusense* MB-17a contains seven genes that are responsible for iron acquisition and its metabolism. These genes encode for the production of the siderophore that helps in iron uptake. Moreover, traits like siderophore and ammonia production were also confirmed by PGP characteristics and were found to help in aerobactin biosynthesis and ammonia assimilation. Aerobactin biosynthesis genes, viz., iucABCD, are either chromosome or plasmid borne and cloned from the plasmid pColV-K30 (de Lorenzo et al., 1986). The strain has 21 genes for membrane transport, with different types of transporters, viz., uni-sym and antiporters, trap transporters, and ABC transporter system, that help in the movement of different types of solutes within the host plant. Apart from this, it also possesses three genes for nitrogen metabolism that help in the assimilation of ${\text{NH}}_{4}^{+}$ during the symbiosis process. Furthermore, the genome of *R. pusense* MB-17a also contains 22 stress responding genes, out of which 10 genes are responsible for the oxidative and 10 for osmotic stress response along with two genes for the detoxification process. The genes responsible for osmotic stresses participate in choline and betaine biosynthesis. These stress response genes like rpoH, otsAB, and clpB enhance the symbiosis process during adverse conditions. Also, glutathione biosynthesis and the gamma-glutamyl cycle are responsible for osmotic tolerance. Along with stress response, eight genes code for the defensive mechanism against various pathogens. Consequently, these genes encode the various mechanisms under stressful conditions.

**Table 7:** Depiction of different genes encoding for PGP traits and their roles.

**S. No**.	**Genes for PGP traits**	**Role**
**1**	**Nitrogen metabolism genes**	
	NRI	Nitrogen regulation protein
	GlxC	Glutamate synthase
	KdpE	DNA binding response regulator
	LysR	Transcriptional regulator
	OmpR	Transcriptional response regulator
**2**	**Genes for defense mechanism**	
	ChrA	Resistance to chromium compounds and various toxic compounds
**3**	**Stress responsive genes**	
	CysA	Act as sulfate and thiosulfate importing ATP binding
	Xre	In transcriptional regulation
	rpoH, otsAB, clpB	In the symbiosis process during adverse conditions
**4**	**Iron acquisition and metabolism genes**	
	FhuA	Act as an outer membrane receptor
	FhuB	Act as ABC transporter
	FhuC	Iron chelator utilization protein
	FhuD	Periplasmic binding substrate protein
**5**	**Genes for membrane transport**	
	DctM	Permease component and decarboxylate transporter
	DctQ	Decarboxylate transporter
	TonB	TRAP transporter
**6**	**Sulfur metabolism genes**	
	DsrE	In galactosylceramide and sulfatide metabolism

In some previous studies, it is reported that genes cysA, cysP, and cysW help in the transportation process of thiosulfate and sulfate in *Pseudomonas* species that act as PGPR. Also, the *otsA*/*otsB* pathway has also been studied under stressful conditions like low temperature, high salinity, and osmotic stress (Duan et al., 2013). Moreover, copA, copB, and copC genes code for copper resistivity by the various strains of PGPR (Voloudakis et al., 2005). Also, betB, betA, and betT act as betaine aldehyde dehydrogenase, choline dehydrogenase, and choline transporter, respectively, which validates the role of *Sphingomonas* species LK11 in PGP traits and also in stress resistance mechanism due to the presence of Na^+^/H^+^ antiporters (nha) (Halo et al., 2015).

## Conclusion

This study presented the multiple PGP traits of *R. pusense* MB-17a that improve and enhance the growth and development of *V. radiata* under different field capacities. A substantial increase in the plant's fresh weight, plant dry weight, nodule number, and nodule fresh weight was confirmed by a pot experiment at the 60th day after the inoculation of *R. pusense* MB-17a. Besides, the genomic annotation of this strain reveals the genes involved in nitrogen metabolism, sulfur metabolism, defense mechanism, iron acquisition and metabolism, and membrane transport. A total of 22 stress responding genes are responsible for the oxidative and osmotic stress response along with the detoxification process, and seven genes are responsible for iron acquisition and its metabolism. Furthermore, it has 21 genes for membrane transport with different types of transporters, viz., uni-sym and antiporters, trap transporters, and ABC transporter system, that help in the movement of different types of solutes within the host plant. Overall, the annotated genome gives information to confirm, identify, and recognize many of the strain's previously assessed properties that are relevant and significant to the promotion of plant growth under various stress conditions. The exploitation of this resourceful PGPR may be an imperative eco-friendly substitute in improving PGP traits, crop growth, and also for phytoremediation under intense environmental conditions.

## Data Availability Statement

The datasets presented in this study can be found in online repositories. The names of the repository/repositories and accession number(s) can be found in the article/supplementary material.

## Author Contributions

PS conceptualized the idea. TC conducted the experimentation, data curation and analysis, original draft preparation under supervision of PS. PS edited the manuscript. PS and RG finally proofread the manuscript. All authors read and approved the manuscript. All authors read and approved the manuscript.

## Conflict of Interest

The authors declare that the research was conducted in the absence of any commercial or financial relationships that could be construed as a potential conflict of interest.

## References

[B1] Abdiev A. Khaitov B. Toderich K. Park K. W. (2019). Growth, nutrient uptake and yield parameters of chickpea (*Cicer arietinum* L.) enhance by *Rhizobium* and *Azotobacter* inoculations in saline soil. J. Plant Nutr. 42, 2703–2714. 10.1080/01904167.2019.1655038

[B2] Alexandre A. Oliveira S. (2013). Response to temperature stress in rhizobia. Crit. Rev. Microbiol. 39, 219–228. 10.3109/1040841X.2012.702097 22823534

[B3] Ali S. Hameed S. Shahid M. Iqbal M. Lazarovits G. Imran A. (2020). Functional characterization of potential PGPR exhibiting broad-spectrum antifungal activity. Microbiol. Res. 232:126389. 10.1016/j.micres.2019.126389 31821969

[B4] Andy A. K. Masih S. A. Gour V. S. (2020). Isolation, screening and characterization of plant growth promoting rhizobacteria from rhizospheric soils of selected pulses. Biocatal. Agric. Biotechnol. 27:101685 10.1016/j.bcab.2020.101685

[B5] Ayuso-Calles M. Garcia-Estevez I. Jimenez-Gomez A. Flores-Felix J. D. Escribano-Bailon M. T. Rivas R. (2020). *Rhizobium laguerreae* improves productivity and phenolic compound content of lettuce (*Lactuca sativa* L.) under saline stress conditions. Foods 9:1166. 10.3390/foods9091166 32847018 PMC7555320

[B6] Aziz R. K. Bartels D. Best A. A. DeJongh M. Disz T. Edwards R. A.. (2008). The RAST Server: rapid annotations using subsystems technology. BMC Genomics 9:75. 10.1186/1471-2164-9-75 18261238 PMC2265698

[B7] Bhattacharyya P. N. Jha D. K. (2012). Plant growth-promoting rhizobacteria (PGPR): emergence in agriculture. World J. Microbiol. Biotechnol. 28, 1327–1350. 10.1007/s11274-011-0979-9 22805914

[B8] Cappuccino J.C. Sherman N. (1992). In: Microbiology: A Laboratory Manual, third ed. Benjamin/cummings Pub. Co., New York, NY, 125–179

[B9] Chawngthu L. Hnamte R. Lalfakzuala R. (2020). Isolation and characterization of rhizospheric phosphate solubilizing bacteria from wetland paddy field of Mizoram, India. Geomicrobiol. J. 37, 366–375. 10.1080/01490451.2019.1709108

[B10] de Lorenzo V. Bindereif A. Paw B. H. Neilands J. B. (1986). Aerobactin biosynthesis and transport genes of plasmid ColV-K30 in *Escherichia coli* K-12. J. Bacteriol. 165, 570–578. 10.1128/JB.165.2.570-578.1986 2935523 PMC214457

[B11] de Souza R. Beneduzi A. Ambrosini A. Da Costa P.B. Meyer J. Vargas L.K. Schoenfeld R. Passaglia L.M. (2013). The effect of plant growth-promoting rhizobacteria on the growth of rice (*Oryza sativa L*.) cropped in southern Brazilian fields. Plant and soil, 366(1-2), 585–603. 10.1007/s11104-012-1430-1

[B12] Dikin A. Sijam K. Kadir J. Seman I.A. (2006). Antagonistic bacteria against Schizophyllum commune Fr. in Peninsular Malaysia. Biotropia 13, 111–121. 10.11598/btb.2006.13.2.221

[B13] Duan J. Jiang W. Cheng Z. Y. Heikkila J. J. Glick B. R. (2013). The complete genome sequence of the plant growth-promoting bacterium *Pseudomonas* sp UW4. PLoS ONE 8:e58640. 10.1371/journal.pone.0058640 23516524 PMC3596284

[B14] Ferreira C. M. H. Soares H. M. V. Soares E.V. (2019). Promising bacterial genera for agricultural practices: an insight on plant growth-promoting properties and microbial safety aspects. Sci. Total Environ. 682, 779–799. 10.1016/j.scitotenv.2019.04.225 31146074

[B15] Frank B. (1889). Ueber die pilzsymbiose der leguminosen. Ber. Deutsch. Bot. Ges. 7, 332–346.

[B16] Garcia-Fraile P. Carro L. Robledo M. Bahena M. H. R. Flores-Felix J. D. Fernandez M. T.. (2012). *Rhizobium* promotes non-legumes growth and quality in several production steps: towards a biofertilization of edible raw vegetables healthy for humans. PLoS ONE 7:38122. 10.1371/journal.pone.0038122 22675441 PMC3364997

[B17] Gopalakrishnan S. Sathya A. Vijayabharathi R. Varshney R. K. Gowda C. L. Krishnamurthy L. (2015). Plant growth promoting rhizobia: challenges and opportunities. 3 Biotech 5, 355–377. 10.1007/s13205-014-0241-x 28324544 PMC4522733

[B18] Gordon A. S. Weber R. P. (1951). Colorimetric estimation of indole acetic acid. Plant Physiol. 26, 192–195. 10.1104/pp.26.1.192 16654351 PMC437633

[B19] Hakim S. Mirza B. S. Imran A. Zaheer A. Yasmin S. Mubeen F.. (2020). Illumina sequencing of 16S rRNA tag shows disparity in rhizobial and non-rhizobial diversity associated with root nodules of mung bean (*Vigna radiata* L.) growing in different habitats in Pakistan. Microbiol. Res. 231:126356. 10.1016/j.micres.2019.126356 31722286

[B20] Halo B. A. Khan A. L. Waqas M. Al-Harrasi A. Hussain J. Ali L. (2015). Endophytic bacteria (*Sphingomonas* sp. LK11) and gibberellin can improve *Solanum lycopersicum* growth and oxidative stress under salinity. J. Plant Interact. 10, 117–125. 10.1080/17429145.2015.1033659

[B21] Hang P. Zhang L. Zhou X. Y. Hu Q. Jiang J. D. (2019). *Rhizobium album* sp. nov., isolated from a propanil-contaminated soil. Anton Leeuw 112, 319–327. 10.1007/s10482-018-1160-3 30178161

[B22] Hossain A. Gunri S. K. Barman M. Sabagh A. E. da Silva J. A. T. (2019). Isolation, characterization and purification of *Rhizobium* strain to enrich the productivity of groundnut (*Arachishypogaea L*.). Open Agric. 4, 400–409. 10.1515/opag-2019-0040

[B23] Igiehon N. O. Babalola O. O. Aremu B. R. (2019). Genomic insights into plant growth promoting rhizobia capable of enhancing soybean germination under drought stress. BMC Microbiol. 19:159. 10.1186/s12866-019-1536-1 31296165 PMC6624879

[B24] Iqbal M. A. Khalid M. Zahir Z. A. Ahmad R. (2016). Auxin producing plant growth promoting rhizobacteria improve growth, physiology and yield of maize under saline field conditions. Int. J. Agric. Biol. 18, 37–45. 10.17957/IJAB/15.0059

[B25] Ji J. Yuan D. Jin C. Wang G. Li X. Guan C. (2020). Enhancement of growth and salt tolerance of rice seedlings (*Oryza sativa* L.) by regulating ethylene production with a novel halotolerant PGPR strain *Glutamici bacter* sp. YD01 containing ACC deaminase activity. Acta Physiol. Plantarum 42, 1–17. 10.1007/s11738-020-3034-3

[B26] Karthika S. Midhun S. J. Jisha M. S. (2020). A potential antifungal and growth-promoting bacterium Bacillus sp. KTMA4 from tomato rhizosphere. Microb. Pathogen. 142:104049. 10.1016/j.micpath.2020.104049 32045643

[B27] King E.J. (1932). The colorimetric determination of phosphorus. Biochemical Journal, 26, 292–297. 10.1042/bj0260292 16744823 PMC1260904

[B28] Kour D. Rana K. L. Yadav A. N. Sheikh I. Kumar V. Dhaliwal H. S. (2020). Amelioration of drought stress in Foxtail millet (*Setaria italica* L.) by P-solubilizing drought-tolerant microbes with multifarious plant growth promoting attributes. J. Environ. Sustain. 3, 23–34. 10.1007/s42398-020-00094-1

[B29] Kremer R. J. Souissi T. (2001). Cyanide production by rhizobacteria and potential for suppression of weed seedling growth. Curr. Microbiol. 43, 182–186. 10.1007/s002840010284 11400067

[B30] Lebrazi S. Fadil M. Chraibi M. Fikri-Benbrahim K. (2020). Screening and optimization of indole-3-acetic acid production by Rhizobium sp. strain using response surface methodology. J. Genet. Eng. Biotechnol. 18, 1–10. 10.1186/s43141-020-00035-9 32562048 PMC7305276

[B31] Lugtenberg B. Kamilova F. (2009). Plant-growth-promoting rhizobacteria. Annu. Rev. Microbiol. 63, 541–556. 10.1146/annurev.micro.62.081307.162918 19575558

[B32] Manasa K. Reddy S. Triveni S. (2017). Characterization of potential PGPR and antagonistic activities of *Rhizobium* isolates from different rhizosphere soils. J. Pharmacogn. Phytochem. 6, 51–54. 10.20546/ijcmas.2017.605.316

[B33] Massa N. Cesaro P. Todeschini V. Capraro J. Scarafoni A. Cantamessa S. (2020). Selected autochthonous rhizobia, applied in combination with AM fungi, improve seed quality of common bean cultivated in reduced fertilization condition. Appl. Soil Ecol. 148:103507 10.1016/j.apsoil.2020.103507

[B34] Mehboob I. Naveed M. Zahir Z. A. Ashraf M. (2012). Potential of rhizobia for sustainable production of non-legumes, in Crop Production for Agricultural Improvement, eds Ashraf M. Ozturk M. Ahmad M. Aksoy A. (Dordrecht: Springer), 659–704. 10.1007/978-94-007-4116-4_26

[B35] Mehta S. Nautiyal C. S. (2001). An efficient method for qualitative screening of phosphate-solubilizing bacteria. Curr. Microbiol. 43, 51–56. 10.1007/s002840010259 11375664

[B36] Mondal H. K. Mehta S. Kaur H. Gera R. (2017). Characterization of abiotic stress tolerant rhizobia as PGPR of mothbean, clusterbean and mungbean grown in hyper-arid zone of Rajasthan. IJBSM. 8, 309–315. 10.23910/IJBSM/2017.8.2.1793

[B37] Moszer I. (1998). The complete genome of *Bacillus subtilis*: from sequence annotation to data management and analysis. FEBS Lett. 430, 28–36. 10.1016/S0014-5793(98)00620-6 9678589

[B38] Mrabet M. Zribi K. Mhadhbi H. Djebali N. Mhamdi R. Aouani M. E. (2011). Salt tolerance of a *Sinorhizobium meliloti* strain isolated from dry lands: growth capacity and protein profile changes. Ann. Microbiol. 61, 361–369. 10.1007/s13213-010-0153-x

[B39] Msimbira L. A. Smith D. L. (2020). The roles of plant growth promoting microbes in enhancing plant tolerance to acidity and alkalinity stresses. Front. Sustain. Food Syst. 4:106 10.3389/fsufs.2020.00106

[B40] Mus F. Crook M. B. Garcia K. Costas A. G. Geddes B. A. Kouri E. D.. (2016). Symbiotic nitrogen fixation and the challenges to its extension to nonlegumes. Appl. Environ. Microbiol. 82, 3698–3710. 10.1128/AEM.01055-16 27084023 PMC4907175

[B41] Olsen S. Sommers L. (1982). Phosphorus, in Methods of Soil Analysis. Part 2. Chemical and Microbiological Properties of Phosphorus, eds Page A. L. Miller R. H. Keeney D. R. (Madison, WI: American Society of Agronomy, Soil Science Society of America), 403–430.

[B42] Overbeek R. Olson R. Pusch G.D. Olsen G.J. Davis J.J. Disz T. Edwards R.A. Gerdes S. Parrello B. Shukla M. Vonstein V. (2014). The SEED and the Rapid Annotation of microbial genomes using Subsystems Technology (RAST). Nucleic acids research, 42(D1),D206–D214. 10.1093/nar/gkt1226 24293654 PMC3965101

[B43] Penrose D. M. Glick B. R. (2003). Methods for isolating and characterizing of ACC deaminase-containing plant growth promoting rhizobacteria. Plant Physiol. 118, 10–15. 10.1034/j.1399-3054.2003.00086.x 12702008

[B44] Pikovskaya R. I. (1948). Mobilization of phosphorous in soil in connection with vital activity of some microbial species. Mikrobiologiya 17, 362–370.

[B45] Poole P. Ramachandran V. Terpolilli J. (2018). Rhizobia: from saprophytes to endosymbionts. Nat. Rev. Microbiol. 16, 291–303. 10.1038/nrmicro.2017.171 29379215

[B46] Pravin V. Rosazlin A. Tumirah K. Ismail S. Boyce A. N. (2016). Role of plant growth promoting rhizobacteria in agricultural sustainability-a review. Molecules 21:573 10.3390/molecules21050573 PMC6273255 27136521

[B47] Premono M. E. Moawad A. M. Vlek P. L. G. (1996). Effect of phosphate-solubilizing Pseudomonas putida on the growth of maize and its survival in the rhizosphere. Crop Sci. 11, 13–23.

[B48] Rana A. Saharan B. Joshi M. Prasanna R. Kumar K. Nain L. (2011). Identification of multi-trait PGPR isolates and evaluating their potential as inoculants for wheat. Ann. Microbiol. 61, 893–900. 10.1007/s13213-011-0211-z

[B49] Schwyn B. Neilands J. B. (1987). Universal chemical assay for the detection and determination of siderophores. Anal. Biochem. 160, 47–56. 10.1016/0003-2697(87)90612-9 2952030

[B50] Souza R. D. Ambrosini A. Passaglia L. M. (2015). Plant growth-promoting bacteria as inoculants in agricultural soils. Genet. Mol. Biol. 38, 401–419. 10.1590/S1415-475738420150053 26537605 PMC4763327

[B51] Suarez C. Ratering S. Hain T. Fritzenwanker M. Goesmann A. Blom J.. (2019). Complete genome sequence of the plant growth-promoting bacterium *Hartmannibacter diazotrophicus* strain E19T. Int. J. Genomics 2019:7586430. 10.1155/2019/7586430 31583244 PMC6754898

[B52] Tang Y.W. Bonner J. (1948). The enzymatic inactivation of indole acetic acid. II. The physiology of the enzyme. Am. J. Bot. 35, 570–578. 10.2307/2438053 18098795

[B53] Tewari S. Sharma S. (2020). Rhizobial exopolysaccharides as supplement for enhancing nodulation and growth attributes of *Cajanus cajan* under multi-stress conditions: a study from lab to field. Soil Tillage Res. 198:104545 10.1016/j.still.2019.104545

[B54] Voloudakis A. E. Reignier T. M. Cooksey D. A. (2005). Regulation of resistance to copper in *Xanthomonasaxonopodispvvesicatoria*. Appl. Environ. Microb. 71, 782–789. 10.1128/AEM.71.2.782-789.2005 15691931 PMC546827

[B55] Wilson K. (2001). Preparation of genomic DNA from bacteria. Curr. Protoc. Mol. Biol. 56, 2–4. 10.1002/0471142727.mb0204s56 18265184

[B56] Yadav A. N. Verma P. Kumar M. Pal K. K. Dey R. Gupta A. (2015). Diversity and phylogenetic profiling of niche-specific Bacilli from extreme environments of India. Ann. Microbiol. 65, 611–629. 10.1007/s13213-014-0897-9

[B57] Yang Y. Zhang S. W. Li K. T. (2019). Antagonistic activity and mechanism of an isolated *Streptomyces corchorusii* stain AUH-1 against phytopathogenicfungi. World J. Microbiol. Biotechnol. 35:145. 10.1007/s11274-019-2720-z 31493267

[B58] Zhang X. X. Gao J. S. Cao Y. H. Sheirdil R. A. Sheirdil X. C. Zhang L. (2015). Isolation and proposal novel rice promoting endophytic bacteria, *Rhizobium oryzicola* sp. nov. Int. J. Syst. Evol. Microbiol. 65:2931. 10.1099/ijs.0.000358 26016492

[B59] Zhao J.-J. Zhang X. Sun L. Zhang R.-J. Zhang C.-W. Yin H.-Q.. (2017). *Rhizobium oryziradicis* sp. nov.isolated from rice roots. Int. J. Syst. Evol. Microbiol. 67, 963–968. 10.1099/ijsem.0.001724 27959784

[B60] Zinina V. V. Korzhenkov A. A. Tepliuk A. V. Kanikovskaja A. A. Patrushev M. V. Kublanov I. V.. (2019). Data on draft genome sequence of *Bacillus* sp. strain VKPM B-3276 isolated from *Culex pipiens* larvae. Data Brief. 24:103757. 10.1016/j.dib.2019.103757 30976634 PMC6441799

